# *N*,*N*′-Dibenzyl-*N*,*N*′-dimethyl-*N*′′-(methyl­sulfon­yl)phospho­ric triamide

**DOI:** 10.1107/S1600536811015832

**Published:** 2011-05-07

**Authors:** Mehrdad Pourayoubi, Sepideh Sadeghi Seraji, Giuseppe Bruno, Hadi Amiri Rudbari

**Affiliations:** a Department of Chemistry, Ferdowsi University of Mashhad, Mashhad 91779, Iran; b Dipartimento di Chimica Inorganica, Vill. S. Agata, Salita Sperone 31, Università di Messina, 98166 Messina, Italy

## Abstract

In the title compound, C_17_H_24_N_3_O_3_PS, the P and the S atoms are each in a distorted tetra­hedral environment and the N atoms display *sp* ^2^ character. The phosphoryl group and the NH unit are *anti* with respect to one another. The dihedral angle between the mean planes of the benzene rings is 31.08 (8)°. The crystal packing is stabilized by N—H⋯O hydrogen bonds, forming an extended chain parallel to the *b* axis.

## Related literature

For phospho­ramidates having a P(O)[N(CH_3_)(CH_2_C_6_H_5_)]_2_ moiety, see: Pourayoubi *et al.* (2010 ▶); Gholivand *et al.* (2005 ▶). For bond lengths in a sulfonamide compound, see: Ibrahim *et al.* (2011 ▶) and references cited therein.
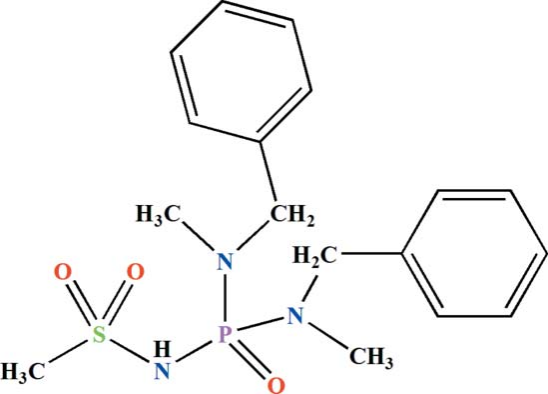


## Experimental

### 

#### Crystal data

C_17_H_24_N_3_O_3_PS
*M* *_r_* = 381.42
Orthorhombic, 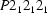

*a* = 8.5343 (4) Å
*b* = 10.1800 (5) Å
*c* = 22.0455 (10) Å
*V* = 1915.29 (16) Å^3^
*Z* = 4
Mo *K*α radiation
μ = 0.27 mm^−1^
*T* = 296 K
0.47 × 0.38 × 0.30 mm

#### Data collection

Bruker APEXII CCD diffractometer
Absorption correction: multi-scan (*SADABS*; Sheldrick, 2004 ▶) *T*_min_ = 0.700, *T*_max_ = 0.746
65627 measured reflections
4155 independent reflections
3937 reflections with *I* > 2σ(*I*)
*R*_int_ = 0.060

#### Refinement

*R*[*F* ^2^ > 2σ(*F* ^2^)] = 0.032
*wR*(*F* ^2^) = 0.094
*S* = 1.08
4155 reflections
226 parameters
H-atom parameters constrained
Δρ_max_ = 0.30 e Å^−3^
Δρ_min_ = −0.22 e Å^−3^
Absolute structure: Flack (1983 ▶), 1769 Friedel pairs
Flack parameter: 0.02 (7)

### 

Data collection: *APEX2* (Bruker, 2005 ▶); cell refinement: *SAINT* (Bruker, 2005 ▶); data reduction: *SAINT*; program(s) used to solve structure: *SHELXS97* (Sheldrick, 2008 ▶); program(s) used to refine structure: *SHELXL97* (Sheldrick, 2008 ▶); molecular graphics: *Mercury* (Macrae *et al.*, 2008 ▶); software used to prepare material for publication: *SHELXTL* (Sheldrick, 2008 ▶) and *enCIFer* (Allen *et al.*, 2004 ▶).

## Supplementary Material

Crystal structure: contains datablocks I, global. DOI: 10.1107/S1600536811015832/jj2084sup1.cif

Structure factors: contains datablocks I. DOI: 10.1107/S1600536811015832/jj2084Isup2.hkl

Additional supplementary materials:  crystallographic information; 3D view; checkCIF report

## Figures and Tables

**Table 1:** Hydrogen-bond geometry (Å, °)

*D*—H⋯*A*	*D*—H	H⋯*A*	*D*⋯*A*	*D*—H⋯*A*
N3—H⋯O1^i^	0.86	1.91	2.7152 (19)	155

Symmetry code: (i) 
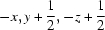
.

**Fig. 1.**
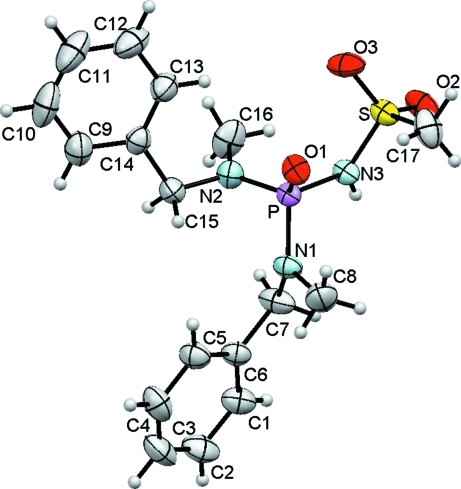
An ORTEP-style plot of title compound with labeling. Ellipsoids are given at the 50% probability level.

**Fig. 2.**
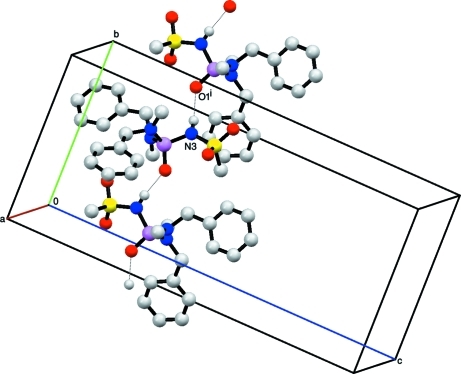
Partial packing view showing the formation of the chain through N—H···O hydrogen bonds which are shown as dotted lines. H atoms not involved in hydrogen bondings have been omitted for the sake of clarity. [Symmetry code: (i) -x, y + 1/2, -z + 1/2]

## supplementary crystallographic information

### Comment

Structure determination of the title compound, CH_3_S(O)_2_NHP(O)[N(CH_3_)(CH_2_C_6_H_5_)]_2_ (Fig. 1), was performed as a part of a project in our laboratory on the synthesis of new phosphoramidate compounds having a P(O)[N(CH_3_)(CH_2_C_6_H_5_)]_2_ moiety (Pourayoubi *et al.*, 2010).

The P═O and P—N bond lengths are comparable to those in similar phosphoramidates of the formula *XP*(O)[N(CH_3_)(CH_2_C_6_H_5_)]_2_ [where *X* = Cl, C_6_H_5_C(O)NH, CCl_3_C(O)NH] (Gholivand *et al.*, 2005). The P—N1 and P—N2 bonds (with bond lengths of 1.6326 (17) Å and 1.6285 (18) Å) are shorter than the P—N3 bond (1.6714 (15) Å). The S═O bond lengths of 1.4317 (16) Å & 1.4287 (18) Å are standard for sulfonamide compounds (Ibrahim *et al.*, 2011).

Each of the phosphorus and sulfur atoms has a distorted tetrahedral configuration. The surrounding bond angles are in the range of 104.84 (9)° to 117.02 (9)° around the P atom and 104.79 (12)° to 118.53 (12)° for the S atom. The average of surrounding angles around the tertiary nitrogen atom N2 (which is about 120°) shows that it is bonded in an essentially planar geometry; whereas, the environment of N1 is slightly deviated from planarity (average of bond angles is 118.3°). Furthermore, the S—N3—P angle is 125.05 (9)°. The dihedral angle between the mean planes of the benzene rings is 31.08 (8)°.

The phosphoryl group and the NH unit adopt the *anti* orientations with respect to each other. Crystal packing is stabilized by N—H···O hydrogen bonds, forming an extended chain parallel to the *b* axis (Fig. 2).

### Experimental

CH_3_S(O)_2_NHP(O)Cl_2_ was synthesized from the reaction between phosphorus pentachloride (16.7 mmol) and methanesulfonamide (16.7 mmol) in dry CCl_4_ at 353 K (3 h) and then treated with formic acid 85% (16.7 mmol) at ice bath temperature.

To a solution of CH_3_S(O)_2_NHP(O)Cl_2_ (2.35 mmol) in dry chloroform (30 ml), a solution of *N*-methylbenzylamine (9.40 mmol) in the same solvent (10 ml) was added at ice bath temperature. After 4 h stirring, the solvent was removed and the product was washed with distilled water and recrystallized from CH_3_CN/CHCl_3_ at room temperature.

### Refinement

H atoms were placed in calculated positions and included in the refinement in a riding-model approximation with CH = 0.93, CH_2_ = 0.97, CH_3_ = 0.96 Å or N—H = 0.86 Å with *U*_iso_(H) parameters equal to 1.5*U*_eq_(C) for methyl H atoms and 1.2*U*_eq_(C,N) for other H atoms.

### Crystal data

**Table:**

C_17_H_24_N_3_O_3_PS	*F*(000) = 808
*M**_r_* = 381.42	*D*_x_ = 1.323 Mg m^−^^3^
Orthorhombic, *P*2_1_2_1_2_1_	Mo *K*α radiation, λ = 0.71073 Å
Hall symbol: P 2ac 2ab	Cell parameters from 9705 reflections
*a* = 8.5343 (4) Å	θ = 2.6–29.5°
*b* = 10.1800 (5) Å	µ = 0.27 mm^−^^1^
*c* = 22.0455 (10) Å	*T* = 296 K
*V* = 1915.29 (16) Å^3^	Irregular, colourless
*Z* = 4	0.47 × 0.38 × 0.30 mm

### Data collection

**Table:**

Bruker APEXII CCD diffractometer	4155 independent reflections
Radiation source: fine-focus sealed tube	3937 reflections with *I* > 2σ(*I*)
graphite	*R*_int_ = 0.060
φ and ω scans	θ_max_ = 27.0°, θ_min_ = 2.2°
Absorption correction: multi-scan (*SADABS*; Sheldrick, 2004)	*h* = −10→10
*T*_min_ = 0.700, *T*_max_ = 0.746	*k* = −13→13
65627 measured reflections	*l* = −28→28

### Refinement

**Table:**

Refinement on *F*^2^	Secondary atom site location: difference Fourier map
Least-squares matrix: full	Hydrogen site location: inferred from neighbouring sites
*R*[*F*^2^ > 2σ(*F*^2^)] = 0.032	H-atom parameters constrained
*wR*(*F*^2^) = 0.094	*w* = 1/[σ^2^(*F*_o_^2^) + (0.0564*P*)^2^ + 0.4498*P*] where *P* = (*F*_o_^2^ + 2*F*_c_^2^)/3
*S* = 1.08	(Δ/σ)_max_ = 0.001
4155 reflections	Δρ_max_ = 0.30 e Å^−^^3^
226 parameters	Δρ_min_ = −0.22 e Å^−^^3^
0 restraints	Absolute structure: Flack (1983), 1769 Friedel pairs
Primary atom site location: structure-invariant direct methods	Flack parameter: 0.02 (7)

### Special details

**Table:**

Geometry. All s.u.'s (except the s.u. in the dihedral angle between two l.s. planes) are estimated using the full covariance matrix. The cell s.u.'s are taken into account individually in the estimation of s.u.'s in distances, angles and torsion angles; correlations between s.u.'s in cell parameters are only used when they are defined by crystal symmetry. An approximate (isotropic) treatment of cell s.u.'s is used for estimating s.u.'s involving l.s. planes.
Refinement. Refinement of *F*^2^ against ALL reflections. The weighted *R*-factor *wR* and goodness of fit *S* are based on *F*^2^, conventional *R*-factors *R* are based on *F*, with *F* set to zero for negative *F*^2^. The threshold expression of *F*^2^ > 2σ(*F*^2^) is used only for calculating *R*-factors(gt) *etc*. and is not relevant to the choice of reflections for refinement. *R*-factors based on *F*^2^ are statistically about twice as large as those based on *F*, and *R*- factors based on ALL data will be even larger.

### Fractional atomic coordinates and isotropic or equivalent isotropic displacement parameters (Å^2^)

**Table:**

	*x*	*y*	*z*	*U*_iso_*/*U*_eq_	
S	−0.14987 (6)	0.66473 (5)	0.33606 (2)	0.03622 (13)	
P	0.01450 (6)	0.58351 (4)	0.22593 (2)	0.02755 (11)	
O2	−0.1835 (2)	0.78944 (16)	0.36311 (8)	0.0531 (5)	
O3	−0.2779 (2)	0.58363 (18)	0.31763 (9)	0.0548 (4)	
O1	0.0074 (2)	0.45080 (12)	0.25313 (7)	0.0376 (3)	
N2	−0.1005 (2)	0.61222 (17)	0.16850 (8)	0.0374 (4)	
N1	0.19197 (19)	0.61606 (16)	0.20271 (8)	0.0357 (4)	
C16	−0.2309 (3)	0.7047 (3)	0.16690 (14)	0.0598 (7)	
H16A	−0.2367	0.7506	0.2049	0.090*	
H16B	−0.3268	0.6577	0.1601	0.090*	
H16C	−0.2149	0.7666	0.1346	0.090*	
C11	−0.4106 (4)	0.2282 (3)	0.07992 (17)	0.0756 (10)	
H11	−0.4817	0.1609	0.0724	0.091*	
C10	−0.3104 (4)	0.2678 (3)	0.03513 (15)	0.0733 (9)	
H10	−0.3123	0.2269	−0.0026	0.088*	
C9	−0.2056 (3)	0.3697 (3)	0.04626 (12)	0.0551 (6)	
H9	−0.1402	0.3989	0.0154	0.066*	
C14	−0.1979 (2)	0.4282 (2)	0.10302 (9)	0.0377 (4)	
C15	−0.0776 (3)	0.5341 (2)	0.11360 (10)	0.0420 (5)	
H15A	0.0251	0.4935	0.1155	0.050*	
H15B	−0.0780	0.5928	0.0789	0.050*	
C7	0.2255 (3)	0.7384 (2)	0.17023 (13)	0.0479 (6)	
H7A	0.2714	0.8007	0.1984	0.058*	
H7B	0.1277	0.7753	0.1558	0.058*	
C6	0.3355 (2)	0.7211 (2)	0.11683 (10)	0.0370 (4)	
C1	0.4198 (3)	0.8292 (2)	0.09625 (11)	0.0451 (5)	
H1	0.4106	0.9094	0.1161	0.054*	
C2	0.5174 (3)	0.8180 (3)	0.04622 (12)	0.0553 (6)	
H2	0.5729	0.8907	0.0324	0.066*	
C3	0.5322 (3)	0.6990 (3)	0.01694 (11)	0.0621 (7)	
H3	0.5980	0.6912	−0.0165	0.074*	
N3	−0.0373 (2)	0.69410 (14)	0.27825 (7)	0.0313 (3)	
H	−0.0016	0.7726	0.2742	0.038*	
C17	−0.0341 (4)	0.5731 (3)	0.38565 (11)	0.0583 (7)	
H17A	0.0536	0.6251	0.3986	0.087*	
H17B	0.0031	0.4958	0.3653	0.087*	
H17C	−0.0952	0.5480	0.4203	0.087*	
C8	0.3248 (3)	0.5572 (3)	0.23501 (12)	0.0588 (7)	
H8A	0.2915	0.4778	0.2546	0.088*	
H8B	0.3631	0.6179	0.2649	0.088*	
H8C	0.4069	0.5375	0.2067	0.088*	
C5	0.3510 (3)	0.6022 (2)	0.08697 (10)	0.0448 (5)	
H5	0.2952	0.5292	0.1002	0.054*	
C4	0.4499 (3)	0.5922 (3)	0.03725 (11)	0.0578 (6)	
H4	0.4604	0.5120	0.0175	0.069*	
C13	−0.2991 (3)	0.3855 (2)	0.14752 (11)	0.0478 (5)	
H13	−0.2951	0.4233	0.1859	0.057*	
C12	−0.4069 (3)	0.2868 (3)	0.13576 (15)	0.0633 (8)	
H12	−0.4766	0.2604	0.1658	0.076*	

### Atomic displacement parameters (Å^2^)

**Table:**

	*U*^11^	*U*^22^	*U*^33^	*U*^12^	*U*^13^	*U*^23^
S	0.0438 (3)	0.0249 (2)	0.0400 (2)	−0.0002 (2)	0.0118 (2)	0.00027 (19)
P	0.0334 (2)	0.01673 (19)	0.0325 (2)	−0.00014 (17)	0.00382 (18)	−0.00090 (16)
O2	0.0683 (12)	0.0342 (8)	0.0568 (9)	0.0053 (8)	0.0255 (9)	−0.0081 (7)
O3	0.0429 (8)	0.0435 (9)	0.0780 (12)	−0.0137 (8)	0.0150 (8)	−0.0002 (8)
O1	0.0544 (8)	0.0169 (6)	0.0414 (7)	−0.0007 (6)	0.0042 (7)	−0.0001 (5)
N2	0.0398 (9)	0.0332 (9)	0.0393 (8)	0.0046 (7)	−0.0017 (7)	−0.0013 (7)
N1	0.0336 (8)	0.0301 (8)	0.0433 (9)	0.0012 (7)	0.0082 (7)	−0.0015 (7)
C16	0.0598 (15)	0.0479 (13)	0.0715 (17)	0.0193 (12)	−0.0217 (14)	−0.0054 (13)
C11	0.083 (2)	0.0558 (17)	0.088 (2)	−0.0198 (16)	−0.0378 (19)	0.0022 (16)
C10	0.092 (2)	0.0602 (17)	0.0673 (18)	0.0035 (17)	−0.0323 (18)	−0.0242 (15)
C9	0.0606 (15)	0.0588 (15)	0.0460 (12)	0.0044 (12)	−0.0048 (11)	−0.0095 (11)
C14	0.0360 (10)	0.0378 (10)	0.0394 (10)	0.0035 (9)	−0.0055 (8)	−0.0014 (8)
C15	0.0426 (11)	0.0498 (12)	0.0335 (10)	−0.0064 (10)	0.0038 (9)	−0.0025 (9)
C7	0.0481 (12)	0.0276 (10)	0.0681 (15)	−0.0014 (9)	0.0255 (12)	−0.0040 (10)
C6	0.0321 (10)	0.0344 (10)	0.0445 (11)	0.0045 (8)	0.0053 (9)	0.0037 (8)
C1	0.0411 (11)	0.0380 (11)	0.0562 (13)	0.0045 (10)	0.0081 (10)	0.0102 (10)
C2	0.0521 (13)	0.0568 (14)	0.0572 (14)	0.0027 (12)	0.0138 (12)	0.0231 (12)
C3	0.0648 (17)	0.0809 (19)	0.0404 (12)	0.0116 (15)	0.0189 (12)	0.0099 (13)
N3	0.0405 (8)	0.0157 (6)	0.0376 (8)	−0.0018 (6)	0.0083 (7)	−0.0012 (6)
C17	0.090 (2)	0.0473 (13)	0.0379 (11)	0.0056 (14)	−0.0010 (12)	0.0081 (10)
C8	0.0402 (12)	0.0805 (19)	0.0558 (14)	0.0078 (13)	−0.0026 (11)	−0.0025 (13)
C5	0.0418 (11)	0.0420 (12)	0.0505 (12)	0.0027 (10)	0.0077 (10)	−0.0021 (9)
C4	0.0680 (16)	0.0596 (16)	0.0456 (12)	0.0113 (13)	0.0119 (11)	−0.0092 (12)
C13	0.0491 (12)	0.0486 (13)	0.0456 (12)	−0.0100 (10)	−0.0059 (9)	0.0022 (10)
C12	0.0523 (15)	0.0623 (17)	0.0751 (18)	−0.0195 (13)	−0.0145 (13)	0.0149 (14)

### Geometric parameters (Å, °)

**Table:**

S—O3	1.4287 (18)	C15—H15B	0.9700
S—O2	1.4317 (16)	C7—C6	1.516 (3)
S—N3	1.6236 (16)	C7—H7A	0.9700
S—C17	1.744 (3)	C7—H7B	0.9700
P—O1	1.4794 (13)	C6—C5	1.384 (3)
P—N2	1.6285 (18)	C6—C1	1.390 (3)
P—N1	1.6326 (17)	C1—C2	1.387 (3)
P—N3	1.6714 (15)	C1—H1	0.9300
N2—C16	1.458 (3)	C2—C3	1.378 (4)
N2—C15	1.461 (3)	C2—H2	0.9300
N1—C7	1.465 (3)	C3—C4	1.369 (4)
N1—C8	1.467 (3)	C3—H3	0.9300
C16—H16A	0.9600	N3—H	0.8600
C16—H16B	0.9600	C17—H17A	0.9600
C16—H16C	0.9600	C17—H17B	0.9600
C11—C12	1.368 (5)	C17—H17C	0.9600
C11—C10	1.367 (5)	C8—H8A	0.9600
C11—H11	0.9300	C8—H8B	0.9600
C10—C9	1.392 (4)	C8—H8C	0.9600
C10—H10	0.9300	C5—C4	1.387 (3)
C9—C14	1.387 (3)	C5—H5	0.9300
C9—H9	0.9300	C4—H4	0.9300
C14—C13	1.378 (3)	C13—C12	1.387 (4)
C14—C15	1.507 (3)	C13—H13	0.9300
C15—H15A	0.9700	C12—H12	0.9300
			
O3—S—O2	118.53 (12)	C6—C7—H7A	108.8
O3—S—N3	109.61 (10)	N1—C7—H7B	108.8
O2—S—N3	106.39 (9)	C6—C7—H7B	108.8
O3—S—C17	107.61 (13)	H7A—C7—H7B	107.7
O2—S—C17	109.08 (12)	C5—C6—C1	119.2 (2)
N3—S—C17	104.79 (12)	C5—C6—C7	121.98 (19)
O1—P—N2	117.02 (9)	C1—C6—C7	118.82 (19)
O1—P—N1	110.51 (9)	C2—C1—C6	120.3 (2)
N2—P—N1	106.20 (9)	C2—C1—H1	119.8
O1—P—N3	108.94 (8)	C6—C1—H1	119.8
N2—P—N3	104.84 (9)	C3—C2—C1	120.0 (2)
N1—P—N3	108.98 (8)	C3—C2—H2	120.0
C16—N2—C15	115.69 (19)	C1—C2—H2	120.0
C16—N2—P	126.49 (17)	C4—C3—C2	119.8 (2)
C15—N2—P	117.76 (15)	C4—C3—H3	120.1
C7—N1—C8	115.7 (2)	C2—C3—H3	120.1
C7—N1—P	120.48 (14)	S—N3—P	125.05 (9)
C8—N1—P	118.83 (16)	S—N3—H	117.5
N2—C16—H16A	109.5	P—N3—H	117.5
N2—C16—H16B	109.5	S—C17—H17A	109.5
H16A—C16—H16B	109.5	S—C17—H17B	109.5
N2—C16—H16C	109.5	H17A—C17—H17B	109.5
H16A—C16—H16C	109.5	S—C17—H17C	109.5
H16B—C16—H16C	109.5	H17A—C17—H17C	109.5
C12—C11—C10	120.5 (3)	H17B—C17—H17C	109.5
C12—C11—H11	119.8	N1—C8—H8A	109.5
C10—C11—H11	119.8	N1—C8—H8B	109.5
C11—C10—C9	119.6 (3)	H8A—C8—H8B	109.5
C11—C10—H10	120.2	N1—C8—H8C	109.5
C9—C10—H10	120.2	H8A—C8—H8C	109.5
C14—C9—C10	120.6 (3)	H8B—C8—H8C	109.5
C14—C9—H9	119.7	C6—C5—C4	119.9 (2)
C10—C9—H9	119.7	C6—C5—H5	120.1
C13—C14—C9	118.5 (2)	C4—C5—H5	120.1
C13—C14—C15	122.87 (19)	C3—C4—C5	120.8 (2)
C9—C14—C15	118.6 (2)	C3—C4—H4	119.6
N2—C15—C14	115.23 (18)	C5—C4—H4	119.6
N2—C15—H15A	108.5	C14—C13—C12	120.8 (2)
C14—C15—H15A	108.5	C14—C13—H13	119.6
N2—C15—H15B	108.5	C12—C13—H13	119.6
C14—C15—H15B	108.5	C11—C12—C13	119.9 (3)
H15A—C15—H15B	107.5	C11—C12—H12	120.0
N1—C7—C6	113.73 (16)	C13—C12—H12	120.0
N1—C7—H7A	108.8		
			
O1—P—N2—C16	114.6 (2)	P—N1—C7—C6	−138.44 (18)
N1—P—N2—C16	−121.5 (2)	N1—C7—C6—C5	24.7 (3)
N3—P—N2—C16	−6.2 (2)	N1—C7—C6—C1	−157.2 (2)
O1—P—N2—C15	−62.24 (18)	C5—C6—C1—C2	0.3 (3)
N1—P—N2—C15	61.66 (17)	C7—C6—C1—C2	−177.8 (2)
N3—P—N2—C15	176.96 (15)	C6—C1—C2—C3	−0.5 (4)
O1—P—N1—C7	175.63 (16)	C1—C2—C3—C4	0.3 (4)
N2—P—N1—C7	47.76 (19)	O3—S—N3—P	−41.70 (16)
N3—P—N1—C7	−64.70 (18)	O2—S—N3—P	−170.99 (13)
O1—P—N1—C8	−30.5 (2)	C17—S—N3—P	73.54 (16)
N2—P—N1—C8	−158.39 (18)	O1—P—N3—S	−25.84 (16)
N3—P—N1—C8	89.15 (19)	N2—P—N3—S	100.16 (13)
C12—C11—C10—C9	−0.9 (5)	N1—P—N3—S	−146.48 (13)
C11—C10—C9—C14	2.4 (5)	C1—C6—C5—C4	0.1 (4)
C10—C9—C14—C13	−1.7 (4)	C7—C6—C5—C4	178.2 (2)
C10—C9—C14—C15	177.4 (2)	C2—C3—C4—C5	0.2 (4)
C16—N2—C15—C14	−72.4 (3)	C6—C5—C4—C3	−0.4 (4)
P—N2—C15—C14	104.8 (2)	C9—C14—C13—C12	−0.3 (4)
C13—C14—C15—N2	−15.1 (3)	C15—C14—C13—C12	−179.4 (2)
C9—C14—C15—N2	165.9 (2)	C10—C11—C12—C13	−1.2 (5)
C8—N1—C7—C6	66.9 (3)	C14—C13—C12—C11	1.8 (4)

### Hydrogen-bond geometry (Å, °)

**Table:**

*D*—H···*A*	*D*—H	H···*A*	*D*···*A*	*D*—H···*A*
N3—H···O1^i^	0.86	1.91	2.7152 (19)	155

Symmetry codes: (i) −*x*, *y*+1/2, −*z*+1/2.

## References

[bb1] Allen, F. H., Johnson, O., Shields, G. P., Smith, B. R. & Towler, M. (2004). *J. Appl. Cryst.* **37**, 335–338.

[bb2] Bruker (2005). *APEX2* and *SAINT* Bruker AXS Inc., Madison, Wisconsin, USA.

[bb3] Flack, H. D. (1983). *Acta Cryst.* A**39**, 876–881.

[bb4] Gholivand, K., Pourayoubi, M., Shariatinia, Z. & Mostaanzadeh, H. (2005). *Polyhedron*, **24**, 655–662.

[bb5] Ibrahim, S., Tahir, M. N., Iqbal, N., Shahwar, D. & Raza, M. A. (2011). *Acta Cryst.* E**67**, o298. 10.1107/S1600536811000365 PMC3051711 21522988

[bb6] Macrae, C. F., Bruno, I. J., Chisholm, J. A., Edgington, P. R., McCabe, P., Pidcock, E., Rodriguez-Monge, L., Taylor, R., van de Streek, J. & Wood, P. A. (2008). *J. Appl. Cryst.* **41**, 466–470.

[bb7] Pourayoubi, M., Tarahhomi, A., Rheingold, A. L. & Golen, J. A. (2010). *Acta Cryst.* E**66**, o2524. 10.1107/S1600536810035725 PMC2983404 21587518

[bb8] Sheldrick, G. M. (2004). *SADABS* Uinversity of Göttingen, Germany.

[bb9] Sheldrick, G. M. (2008). *Acta Cryst.* A**64**, 112–122. 10.1107/S0108767307043930 18156677

